# Functional magnetic resonance and swallowing: critical literature review^☆^^☆☆^

**DOI:** 10.1016/j.bjorl.2015.08.006

**Published:** 2015-09-07

**Authors:** Maíra Santilli de Lima, Laura Davison Mangilli, Fernanda Chiarion Sassi, Claudia Regina Furquim de Andrade

**Affiliations:** a Speech Therapy in Orofacial Functions, Speech Therapy Support Unit, Instituto Central do Hospital de Clínicas, Faculdade de Medicina, Universidade de São Paulo (HCFM-USP), São Paulo, SP, Brazil; b Sciences, Faculdade de Medicina, Universidade de São Paulo (USP), São Paulo, SP, Brazil; c Rehabilitation Science Medical Investigation Laboratory, Hospital das Clínicas, Faculdade de Medicina, Universidade de São Paulo (HCFM-USP), São Paulo, SP, Brazil; d Department of Physical Therapy, Speech Therapy and Occupational Therapy, Faculdade de Medicina, Universidade de São Paulo (FM-USP), São Paulo, SP, Brazil; e Faculdade de Medicina, Universidade de São Paulo (FM-USP), São Paulo, SP, Brazil

**Keywords:** Deglutition, Deglutition disorders, Magnetic resonance imaging, Deglutição, Transtornos de deglutição, Imagem por ressonância magnética

## Abstract

**Introduction:**

Aspects of the neuroanatomical representation of swallowing have been investigated in humans through brain mapping techniques, such as functional magnetic resonance imaging (fMRI).

**Objective:**

This critical qualitative review of the literature analyzed international scientific publications in the PubMed database that investigated the activation of the central nervous system in humans during the act of swallowing.

**Methods:**

This investigation was limited to articles that investigated adults older than 18 years, published in English or Portuguese, between January 2002 and December 2013. Publications that did not have access to the full text, that were repeated by overlapping keywords, case studies, literature reviews, letters to the editor, and those not directly related to the topic of the investigation were excluded.

**Results:**

A total of 649 articles were identified, of which 21 matched the inclusion criteria.

**Conclusion:**

The main purpose of the manuscripts that investigate the swallowing process through fMRI were: to characterize swallowing in different pathologies; to compare swallowing in different age groups; to describe brain activation in different stimulation conditions. These studies indicate multiple cortical regions involved in swallowing control. Overall, the studies indicate that fMRI is a non-invasive and quantitative method that allows the investigation of characteristics that are quite often not clinically visible.

## Resumo

**Introdução:**

Aspectos da representação neuroanatômica do funcionamento cortical que controla a deglutição têm sido investigados e identificados em humanos, utilizando-se técnicas de mapeamento cerebral, como a Ressonância Magnética funcional (RNMf).

**Objetivo:**

Esta revisão crítica qualitativa da literatura levantou publicações científicas sobre a funcionalidade do sistema nervoso central durante tarefas de deglutição, por meio da base de dados PubMed.

**Método:**

O levantamento realizado limitou-se a seres humanos, com idade superior a 18 anos, nos idiomas inglês e português, entre janeiro de 2002 e dezembro de 2013. Publicações sem acesso completo, repetidas por sobreposição das palavras chave, estudos de caso, revisões de literatura, cartas ao editor e não relacionadas diretamente ao tema foram excluídas.

**Resultado:**

Identificou-se 649 estudos, sendo que somente 21 se enquadraram aos critérios estabelecidos.

**Conclusão:**

Foram encontrados artigos que objetivaram, por meio da RNMf, estudar a função de deglutição em patologias; comparar a deglutição em diferentes idades; descrever a ativação cerebral em diferentes situações de estimulação. Estes estudos apontam múltiplas regiões corticais que participam no controle da deglutição. Conclui-se que a RNMf é um método não invasivo, quantitativo, que demonstra respostas, muitas vezes, não visíveis clinicamente.

## Introduction

Swallowing is a complex sensory-motor process that involves several physiological stages.^1^ The hypothesis has been proven that multiple brain areas are activated during swallowing, both in children and adults, reflecting regions that are responsible for different aspects of the swallowing process.^2^

It is known that the cerebral cortex plays an important functional role in the regulation of swallowing.^3^ The reflexive components of swallowing depend on the brainstem swallowing centers; the onset of swallowing is a voluntary action, which depends on the integrity of the motor areas of the cortex.^4^

Some aspects of the neuroanatomical representation of cortical function that controls swallowing have been investigated and identified in humans, using brain imaging techniques such as the functional magnetic resonance imaging (fMRI), considered one of the most recent and advanced methods of functional neuroimaging without the use of ionizing radiation.^5^

The advent of functional magnetic resonance imaging has facilitated or a better detection and quantification of organizational changes in cortical activation, with improved spatial and temporal resolution.^5^ fMRI is a safe and non-invasive method to investigate the human brain, and has been indicated in the investigation of dysphagia after brain damage.^6^

The aim of this study was to perform a critical analytic summary of relevant articles on the fMRI findings during swallowing in different groups studied in the international literature.

## Methods

Since this was a non-experimental study, there was no need for consent forms or institutional ethics committee approval. The Cochrane Handbook precepts were followed to establish the research method.^7^

The articles used in this study were selected through the PubMed database using the keywords: “deglutition”, “deglutition disorders”, “magnetic resonance”, and “magnetic resonance spectroscopy”, limited to studies performed in adult individuals, published in English and Portuguese, carried out from January 2002 to December 2013.

The search for publications in the database was independently conducted by the researchers to minimize possible citation losses. Each article recovered from the database was independently analyzed by the researchers to determine its relevance for selection and inclusion in the study. Articles in languages other than Portuguese and English were excluded, as well as publications that did not allow access to the full text (obtained from the CAPES Journal Portal) and those repeated due to overlapping keywords. Of the full texts obtained, those related to case studies, literature reviews, studies with animals, letters to the editor, and those that were not directly related to the topic of investigation were excluded. Texts that were effectively related to the research topic were analyzed. All stages of the study were independently conducted by the researchers. In case of disagreement among the researchers, only texts with a consensual final opinion were included. Due to its nature, this was not a single-blind study.

After crossing “deglutition × magnetic resonance”, “deglutition × magnetic resonance spectroscopy”, “deglutition disorders × magnetic resonance”, and “deglutition disorders × magnetic resonance spectroscopy”, a total of 649 articles were found, of which 151 had unavailable summaries. Of the remaining 498, 189 were repeated. Thus, 309 articles were evaluated and of these, only 21 were included in this study.

The reason for the exclusion of 288 articles from the study is shown in Table 1. The 21 selected articles were critically evaluated regarding the following: objectives; number and gender of participants; age range; criteria and evaluation methods; results; and conclusions.

**Table 1:** Reasons for article exclusion.

Reason for exclusion	Number of excluded articles
Use of the test for diagnosis	155
Use of non-functional magnetic resonance	68
Use in other pathologies/clinical pictures (not dysphagia)	37
Dealt with other tests (not fMRI)	13
Animal studies	5
Literature review	5
Evaluated exam technique	2
Dysphagia study without fMRI	2
Exam used for surgical assistance	1

fMRI, functional magnetic resonance imaging.

## Results

The study results are briefly described in Table 2.

**Table 2:** Summary of the articles used in this review.

Article	Objective	Study sample	Method	Results and Conclusions
Suzuki et al., 2003^8^	Investigate cerebellar and basal ganglia activation during swallowing.	11 right-handed volunteers (24–42 years).	Individuals should swallow the saliva or not, according to verbal commands (“swallow” and “stop”).	Spontaneous swallowing involved the basal ganglia and cerebellum as well as cortical structures.
Martin et al., 2004^9^	Clarify the role of different brain areas in swallowing.	14 right-handed volunteers (mean age of 28 years, standard deviation 6.5).	Participants should perform three different sequences of tasks: (1) saliva swallowing, voluntary elevation of tongue, and voluntary finger opposition; (2) Swallowing saliva, swallowing saliva with effort, and finger opposition; (3) swallowing saliva, voluntary apnea, and finger opposition.	This finding suggests that these areas of the brain may mediate specific processes for swallowing. Approximately 60% of subjects had strong functional lateralization of the postcentral gyrus toward the left hemisphere to swallow, while 40% showed a similar activation trend for the tongue elevation task. This finding supports the view that the oral sensorimotor cortex in the left and right hemispheres are not functionally equivalent.
Mosier et al., 2005^10^	Determine the cortical adaptation mechanisms of swallowing after partial glossectomy (reconstruction with primary closure).	Four patients with partial glossectomy T2 and T3 (mean of 63 years). Eight healthy volunteers (mean 38 years).	Participants had to swallow saliva and water, according to the protocol, after verbal commands.	The adaptive responses involve cortex areas associated with tongue movement planning during swallowing. This reflects adaptive biomechanical processes of tongue movement during swallowing and tongue sensitivity alterations were not verified.
Toogood et al., 2005^11^	Compare brain activation after visual command to swallow and not to swallow.	Eight healthy individuals (mean of 23.8 years, standard deviation of 2.3).	Participants had to swallow their own saliva or not, according to visual stimuli for the commands (“swallow”, “do not swallow”, intercalated with “relax”).	It was concluded that the “swallow” × “do not swallow” paradigm is an effective method to differentiate cortical areas of swallowing processing.
Shibamoto et al., 2007^12^	Elucidate the cortical representations of the oral and pharyngeal stages of swallowing, with different types of food bolus.	21 healthy right-handed adults (mean age of 29.2 years, ranging from 23 to 38).	Participants had to swallow, in the supine position, 5 mL of water at 15 °C, a capsule, and agar (solid) without aid of water and without chewing.	The size of the activated area during the swallowing of water was higher than for agar. The task of swallowing a capsule may require more from oral swallowing and motor coordination than that for solid or liquid food. Cortical representations of swallowing vary by type of food and can explain the responses of variables in the performance of swallowing in patients with oropharyngeal dysphagia.
Martin et al., 2007^13^	Assess the neural representations of voluntary swallowing in healthy elderly.	Nine healthy elderly women (mean 74.2 years, standard deviation 8.1)	Participants had to swallow the saliva accumulated in the mouth or 3 mL of water injected into the oral cavity, in accordance with the visual command.	There was a four-fold increase in the volume of cerebral activation when swallowing water compared to saliva swallowing, particularly the right premotor cortex and the prefrontal cortex. This specific activation pattern may represent a compensatory response to the demands of swallowing water considering the age-related decrease in oral sensorimotor function.
Lowell et al., 2008^14^	Differentiate the motor, sensory planning, and components of motor performance of the cerebral control of swallowing.	14 healthy individuals, only one left-handed (mean age of 36 years, standard deviation of 10.4, ranging from 21 to 52 years).	Four conditions were employed during fMRI: oral sensory stimulation (air pulse), direct swallowing, indirect swallowing, and apnea. During the examination, the individuals were asked to perform tasks after receiving a visual stimulus – symbol: (1) a tube for airflow – oral-sensory stimulation, (2) a lamp – for indirect swallowing, (3) a glass – for direct swallowing and (4) lungs with an X-ray – for apnea.	Sensory oral stimulation and indirect swallowing showed activation in neural areas related to areas activated during spontaneous swallowing. It is suggested that oral sensory stimulation and indirect swallowing have important implications for intervention in swallowing disorders associated with neurological diseases.
Humbert et al., 2009^15^	To assess the functional alterations in swallowing according to age.	11 elderly individuals (mean age of 72.3 years, standard deviation of 7.5, ranging from 64 to 83 years). 12 young individuals (mean age of 27.9 years, standard deviation 4, ranging from 23 to 37 years).	Participants had to swallow saliva, water, and barium, alternating with pauses in swallowing, according to the commands.	The findings suggest that the elderly individuals showed a higher cortical involvement to complete the same swallowing tasks when compared to the younger ones. The elderly showed a higher latency to the onset of pharyngeal swallowing and increased residue of the ingested material in the pharynx. These findings suggest that the elderly had a higher cortical involvement to complete the same swallowing tasks than younger individuals.
Kaway et al., 2009^16^	To investigate brain activation after simultaneous display of stimuli associated with swallowing movements.	12 healthy and right-handed adults, with vision and hearing within the normal range (20–28 years).	Auditory and visual stimuli of the act of swallowing and neck anatomy were presented in association during swallowing. The cortical areas activated with different types of stimuli were analyzed.	According to this study, the audiovisual stimuli associated with the swallowing movement can be applied in the treatment of patients with dysphagia. The activation of areas associated with the swallowing movement (planning and performance) occurred during all stimuli.
Li et al., 2009^17^	To explore the structural and functional changes in patients with amyotrophic lateral sclerosis (ALS), with or without dysphagia, compared to healthy adults.	ALS patients, five with dysphagia and five without dysphagia (32–58 years).	Participants should swallow their own saliva or stop the action, according to visual commands.	During voluntary swallowing of saliva, the preeminent focal activation corresponded to the same cortical area in both groups; however, a decrease in activation was observed in ALS patients with dysphagia
		Ten individuals matched for gender and age to the study group.		Combined, non-invasive neuroimaging techniques may be useful tools for assessing rehabilitation, prognosis, and study strategies for dysphagic patients with ALS, particularly for patients that have ‘negative’ MRI by conventional methods.
Li et al., 2009^18^	To investigate neurorehabilitation mechanisms after dysphagia in patients with unilateral stroke, when compared to healthy adults.	Ten patients after ischemic stroke with severe dysphagia for at least three days (mean age of 70.9 years, standard deviation of 3.4, ranging from 62 to 78 years). Ten healthy elderly individuals (mean age 70.3 years, standard deviation 4.2, ranging from 65 to 75 years).	Participants had to swallow their own saliva or stop the action, according to visual commands.	The results indicate that a unilateral stroke of either cerebral hemisphere may cause dysphagia and recovery of this symptom may be associated with the fact that other unaffected areas start being activated during swallowing.
Malandraki et al., 2009^19^	To identify neural activations of different cerebral components of swallowing in healthy young adults.	Ten healthy young individuals (mean 21.7 years, standard deviation of 2).	Water was injected into the oral cavity of the participants, who should follow random visual commands (“swallow”, “get ready to swallow”, “touch your tongue”, “clear your throat”).	Areas activated during each of the task components showed partial differentiation of neural location for the several components of swallowing. The study was able to identify the brain areas involved in the swallowing task, in agreement with previous findings.
Malandraki et al., 2010^20^	To assess age differences in patterns of neural lateralization during swallowing.	Ten young right-handed individuals (mean age of 21.7 years, standard deviation of 2.1). Nine adults (mean age 70.2 years, standard deviation 3.9)	Water was injected into the oral cavity of the participants, who should follow the commands that appeared on a television at random (“swallow”, “get ready to swallow”, “touch your tongue”, “clear your throat”).	With increasing age, the hemispheric cortical control of swallowing seems to become more symmetrical/bilateral, which may indicate compensatory neural mechanisms of brain aging, commonly seen in other motor and cognitive functions.
Haupage et al., 2010^21^	To correlate CNS adaptation mechanisms with changes in language function.	Six individuals diagnosed with tongue cancer (mean age of 50.8 years – 21–66). Nine healthy individuals (mean age 35.6; 30–48).	The participants were evaluated prior to surgery and after six months through fMRI, with three tasks: “tongue touch”, dry swallowing and water swallowing after auditory command, comparing the inter-individual activation areas (pre and postoperative), preoperative patient with control, and postoperative with control.	Patients who recovered tongue function after partial glossectomy show adaptive responses in the CNS. The increased cortical activation areas after surgery are involved with the planning, the movement and sensation of the tongue during swallowing. Postoperative activation patterns were closer to control levels than to the preoperative exams.
Babaei et al., 2010^22^	To test whether activity of the cortical swallowing network can be increased by sensory stimuli related to food.	14 healthy and right-handed individuals (mean age of 28 years, standard deviation of 10).	The subjects received different visual and olfactory commands to perform saliva or food swallowing (water, lemonade, milk chocolate, and popcorn-flavored solution) at same temperature.	Signal intensity along all sub-cortical regions of the swallowing network significantly increased with stimulation with flavor, when compared with swallowing of saliva and water. The simultaneous olfactory, gustatory, and visual stimulation of ingested substances increases the activity of the swallowing cerebral cortical network. This increase in activity may have implications in the management of dysphagia.
Humbert et al., 2010^23^	To compare the neural activation and the physiology of swallowing in patients with newly diagnosed Alzheimer's disease (AD) and healthy individuals matched for age.	13 Alzheimer patients (mean 74.3 years, standard deviation 8.6, ranging from 58 to 88). 11 healthy individuals (mean age 72.3 years, standard deviation 7.5, ranging from 64 to 83).	Barium and water were injected into the oral cavity of the patients and subjects should swallow when they feel the entire content in their mouth; as well as saliva when they saw the visual command.	The group with AD had significantly lower response in many cortical areas that are traditionally involved in normal swallowing. They did not recruit new regions, or compensate in regions that are normally activated during swallowing. Although swallowing disorders are usually observed in the final stages of AD, changes in the cortical control of swallowing can start long before dysphagia become apparent.
Humbert et al., 2011^24^	To assess brain responses in swallowing tasks, comparing individuals by age and the presence of Alzheimer's disease (AD), through fMRI.	13 elderly individuals with Alzheimer's (mean of 74.3 years, standard deviation 8.6 years – range: 58–88 years). 12 healthy young individuals (mean age 27.9 years, standard deviation 4.0 years – range: 23–37 years) 11 healthy elderly individuals (mean 72.3 years, SD 7.5, range: 58–88 years).	Swallowing of saliva, water, and barium, intercalated with moments of pause in the swallowing task after predetermined commands.	Absence of significant differences in areas of brain activation when comparing elderly individuals, young individuals, and elderly individuals with AD. Individuals with AD required greater effort to perform the pause function during swallowing when the command for this action was requested.
Stice et al., 2011^25^	Compare brain activation during food receiving tasks and receiving monetary reward in young individuals with high and low risk for obesity.	60 young individuals of normal weight, with high and low risk for obesity according to their family members (mean of 15 years, standard deviation 2.9). Of these, 35 had high risk for obesity.	The young individuals were given two stimuli during the examination: (1) stimulus with food reward: after the representation of images – a milkshake or water – received the respective food. This stimulus occurred after a period of fasting to induce hunger; (2) stimulus with monetary reward: during a game, the participant received “money” as a reward (figuratively).	Individuals with high risk for obesity show higher cortical activation when receiving the milkshake, compared to low-risk ones. These young individuals show high responsiveness to circuits connected to reward, in general, with high responsiveness in the somatosensory region connected to food, which can lead to overeating that result in “blunting” of dopamine signaling and high responsiveness to food stimuli.
Malandraki et al., 2011^2^	To determine whether swallowing events recruit different areas of activation, with different amplitudes, comparing young and elderly individuals.	Nine right-handed elderly individuals (mean 70.2 years, standard deviation 3.9). Ten healthy young individuals (mean 21.7 years, standard deviation 2.1).	Water was injected into the oral cavity of the participants, which should follow the commands that appeared on a television at random (“swallow”, “get ready to swallow”, “touch with your tongue”, “clear your throat”).	There was decreased activation in the elderly compared to young individuals during swallowing and the analyzed tasks. These reductions were significant in several primary somatosensory areas, indicating a decline in neural processing of sensory signals to coordinate the swallowing response.
Babaei et al., 2012^26^	To investigate the reproducibility of positive and negative cortical activity (BOLD) related to swallowing in different sessions of fMRI in regions of interest previously demonstrated in other studies.	16 right-handed asymptomatic adult individuals aged 20–34 years, of whom nine were women.	A projection screen was placed in front of the scanner to display visual cues (verbal command) to swallow and/or the image of a cross for ocular fixation between swallows (3s). Two separate sessions were carried out.	All individuals had significant cortical activity within the known areas of the swallowing network in both sessions. The cross-correlation coefficient of the fMRI signal percentage changes and the number of voxels activated through the positive and negative BOLD networks were similar between the two moments. The map of the cortical activity group, as well as length and amplitude of activity induced by spontaneous swallowing in the swallowing cortical network, were reproducible among the studied sessions.
Babaei et al., 2013^27^	To determine the functional connectivity (FC) between brain regions involved in swallowing and characterize the differences in FC between these regions when involved in different tasks (rest or control task).	16 right-handed, adult, asymptomatic individuals aged 20–34 years, of which nine were women.	First the individuals had swallowing analyzed according to a protocol and then, with the aid of a screen for visual projection of commands, three different conditions were assessed: (1) swallowing task after 21 random commands to swallow; (2) ocular fixation on the image of a cross with 21 commands to relax substituting the ocular fixation mark; (3) wakefulness with eyes closed at rest.	Functional connectivity of the swallowing network regions is robust and reproducible across different experimental conditions and is significantly higher during swallowing task when compared to the control of visual task or rest.

SD, standard deviation; fMRI, functional magnetic resonance imaging; AD, Alzheimer's disease; CNS, central nervous system; TV, television; pre-op, preoperative period; post-op, postoperative period; ALS, amyotrophic lateral sclerosis; CVA, cerebrovascular accident.

## Discussion

Thirteen different groups of authors were identified. There was no prevalence or differentiation of results between genders. All items had quantitative analysis, supported by statistical results. Of the 21 selected articles, only one^23^ included the participation of raters, to give reliability to the videofluoroscopic evaluation.

Of the assessed articles, nine^2^, ^10^, ^15^, ^17^, ^18^, ^19^, ^20^, ^21^, ^22^, ^23^, ^24^ used control groups to make comparisons. In 20 articles,^2^, ^8^, ^9^, ^10^, ^11^, ^12^, ^13^, ^14^, ^15^, ^16^, ^17^, ^18^, ^19^, ^20^, ^21^, ^22^, ^23^, ^24^, ^25^, ^26^, ^27^ the study design was cross-sectional, with evaluation/performance of a single examination, and in only one^21^ the design was longitudinal, including the evaluation/performance of examination at two different moments, i.e., pre- and post-operatively. According to the methodological design of this study, all articles used the fMRI exam, but other exams were also used in association, such as electromyography (EMG) and videofluoroscopic swallowing study (VFSS).

The parameters considered for evaluation during the fMRI examination were not consistent. Some studies evaluated the role of swallowing saliva, water, and barium^15^, ^16^, ^23^, ^24^; others, the swallowing of saliva and/or water^2^, ^9^, ^10^, ^13^, ^17^, ^18^, ^19^, ^20^, ^21^; whereas another assessed the swallowing of solids, liquids, and semisolids to differentiate consistencies.^12^ Regarding the instructions to perform the tasks, verbal commands were used, such as “swallow” and “do not swallow”,^8^, ^11^ as well as the visual and/or auditory and/or gustatory stimulation.^14^, ^22^, ^25^, ^26^, ^27^

In general, to analyze the results of the fMRI examination, the blood oxygenation level dependent (BOLD), regions of interest (ROI), cortical lateralization, and diffusion tensor imaging (DTI) criteria were used, as well as time latency.

After a critical review of the articles, it was observed that studies that presented the findings of fMRI during swallowing tasks can be divided into three groups: studies describing brain activation in healthy individuals in different conditions and with different stimuli^8^, ^11^, ^12^, ^13^, ^14^, ^16^, ^19^, ^22^, ^26^, ^27^; studies of patients with diseases/comorbidities, who were compared to healthy individuals,^10^, ^17^, ^18^, ^21^, ^23^, ^24^, ^25^ with the following comorbidities: Alzheimer's disease (AD),^23^, ^24^ obesity,^25^ tongue cancer,^10^, ^21^ amyotrophic lateral sclerosis (ALS),^17^ and ischemic cerebrovascular accident (CVA)^18^; and studies comparing brain activation at different ages.^2^, ^15^, ^20^, ^24^

For better presentation and discussion of the study findings, thematic agglutination was performed according to the abovementioned groups. In cases of AD, in brief, significantly lower response (BOLD) was observed in several cortical areas that are traditionally involved in normal swallowing. One of the studies^23^ concluded that, while the swallowing disorder is generally observed in the final stages of AD, changes in cortical control of swallowing can start long before the dysphagia becomes apparent.

Studies of patients with tongue cancer^10^, ^21^ sought to determine the adaptation mechanisms of the nervous system, after the glossectomy. The fMRI findings of glossectomized patients showed greater activation in the parietal cortex and adaptive responses (in cortex areas associated with tongue movement planning during swallowing) of the CNS after glossectomy.

The study that explored the structural and functional changes in patients with amyotrophic lateral sclerosis, with or without dysphagia^17^ found that, during voluntary saliva swallowing, all individuals had the same activated cortical area, but the dysphagic patients showed a decrease in activation. It was also demonstrated that, even in cases in which the disease cannot be detected by conventional MRI, fMRI may disclose the alterations in brain function during swallowing, showing possible swallowing disorders that may develop in the future.

The findings of articles^8^, ^9^, ^11^, ^12^, ^13^, ^14^, ^16^, ^19^, ^22^, ^26^, ^27^ that only studied brain activation in healthy patients in the presence of different stimuli – consistency, flavor, different instructions to perform the task of swallowing, different ages, and different central nervous system foci – showed the following: (1) higher brain activation: during liquid swallowing compared to solids; during water swallowing compared to saliva swallowing; when the food offered had flavor, compared to saliva and water; when there was a verbal command to perform swallowing; when using olfactory, gustatory, and visual stimuli associated with swallowing; and when using sensory stimulation with air injection in the oral cavity during the swallowing (compared to the times in which there was no injection of air); (2) increased activation in areas of interest (programming and performance) when visual and auditory stimuli associated with swallowing were used in combination to carry out the function; (3) that the oral sensorimotor cortex in the left and right hemispheres are not functionally equivalent; and (4) that spontaneous swallowing involves the activation of the cerebellum and basal ganglia, as well as cortical structures.

By analyzing the normal standards for brain activation during swallowing tasks, it was observed that although the contrast analysis failed to identify the specific activation foci for swallowing, overlapping activation maps suggest that the most lateral extent of the precentral and anterior parietal cortex, rostral anterior cingulate cortex, precuneus and left parietal operculum are preferentially activated in swallowing.^9^ Suzuki et al. (2003),^8^ however, found that regions activated during swallowing were observed in the sensorimotor cortex, insula, cerebellum, putamen, globus pallidus, thalamus, anterior cingulate gyrus, supplementary motor area, superior temporal gyrus, and in the substantia nigra; the cerebellum was bilaterally activated, especially on the left side; activation of the globus pallidus and putamen was bilateral.

In the past few years, a growing number of neuroimaging studies in healthy subjects have shown multiple cortical regions involved in the control of swallowing. Functional brain imaging may help to elucidate the relevant neural mechanisms, identifying the neural patterns that control this complex sensory-motor action, providing evidence of functional changes in the cerebral cortex after a comorbidity, such as those cited in this review, helping to identify and the correct rehabilitation of dysphagia.

This critical review showed that the examination is of great importance in the early identification of brain alterations, facilitating the choice of a better rehabilitation approach for dysphagic patients, or those at risk for dysphagia, allowing improvement of the clinical picture or risk prevention.

## Conclusion

This study demonstrated that the fMRI is a non-invasive, quantitative method that provides specific answers, which are sometimes not clinically identified.

It can be used associated with and/or in addition to other imaging tests to confirm results or as a validation method. As a negative aspect, this study showed that for all examinations, all patients should swallow in the supine position, which may influence the adequate performance of the swallowing function. Another aspect that must be considered is that there was no standardization of the methodologies used in the assessed articles, both in case selection and in the study materials and methods.

## Conflicts of interest

The authors declare no conflicts of interest.
